# Timing of insertable cardiac monitor implantation after embolic stroke of undetermined source and its impact on atrial fibrillation detection: A target trial emulation analysis

**DOI:** 10.1177/17474930261438742

**Published:** 2026-03-24

**Authors:** Lucio D’Anna, Fionn Maguidhir, Robert Simister, Arvind Chandratheva, Gaurav Desai, Maryam Haneef, Anne Idian, Francesco Favruzzo, Alessandra Pes, Claudio Baracchini, Diletta Rosin, Mariarosaria Valente, Gian Luigi Gigli, Liqun Zhang, Nathan Leung Mres, Manav Sohal, Simona Sacco, Raffaele Ornello, Federico De Santis, Ubaldo Coppola, Gabriele Prandin, Selina Edwards, Ceylan Safak, Roberto Avila, Joan Cruz, Ashley Laurie, Michele Romoli, Valentina Tudisco, Federica Nicoletta Sepe, Jianqun Guan, Asha Barnard, Lydia Jeffrey, Jake Dagan, Tsering Dolkar, Jonathan Hayton, Soma Banerjee, Matteo Foschi, Giovanni Merlino, Phang Boon Lim

**Affiliations:** 1 Department of Stroke and Neuroscience, Charing Cross Hospital, Imperial College London NHS Healthcare Trust, London, UK; 2 Department of Brain Sciences, Imperial College London, London, UK; 3 Comprehensive Stroke Service, University College London Hospital, London, UK; 4 Stroke Unit and Neurosonology Laboratory, Padua University School of Medicine, Padua, Italy; 5 Clinical Neurology, Udine University Hospital and Department of Medicine (DAME), University of Udine, Udine, Italy; 6 SOSD Stroke Unit, Department Head, Neck, and Neurosciences, Udine University Hospital, Udine, Italy; 7 Department of Neuroscience, George’s University of London, Stroke, London, UK; 8 Department of Cardiology, St George’s University Hospital NHS Foundation Trust, London, UK; 9 Department of Biotechnological and Applied Clinical Sciences, University of L’Aquila, L’Aquila, Italy; 10 Department of Neuroscience, Neurology and Stroke Unit, Bufalini Hospital, Cesena, Italy; 11 Department of Cardiology, Hammersmith Hospital, Imperial College London NHS Healthcare Trust, London, UK; 12 Department of Neurosciences, Neurology Unit, S. Maria delle Croci Hospital, AUSL Romagna, Ravenna, Italy

**Keywords:** ESUS, atrial fibrillation, ICM, timing

## Abstract

**Background:**

A substantial proportion of ischemic strokes remain classified as embolic stroke of undetermined source (ESUS) despite standard diagnostic evaluation. Prolonged cardiac monitoring with implantable cardiac monitors (ICMs) increases atrial fibrillation (AF) detection, but the optimal timing of ICM implantation after ESUS remains uncertain.

**Aims:**

To evaluate whether early versus delayed ICM implantation after ESUS influences AF detection and time to diagnosis.

**Methods:**

We conducted a multicenter observational cohort study emulating a target trial. Consecutive ESUS patients undergoing ICM implantation were classified as ICM_EARLY_ (⩽30 days) or ICM_DELAYED_ (31–365 days) implantation after the index event. Inverse probability weighting was applied to adjust for baseline confounding. Primary and secondary outcomes included AF detection within 30, 90, and 120 days after implantation, assessed using weighted logistic regression, Poisson models for detection rates per person-time, Cox proportional hazards models, and restricted mean survival time (RMST). Sensitivity analyses included center-level clustering and competing-risk models.

**Results:**

Among 333 patients (90 ICM_EARLY_ and 243 ICM_DELAYED_), early implantation was associated with significantly higher AF detection within 30 days (7.8% vs 1.6%; odds ratio (OR) = 4.49, 95% confidence interval (CI) = 1.17–17.27; p = 0.028) and higher detection rates per person-time (incidence rate ratio (IRR) = 4.26, 95% CI = 1.16–15.60; p = 0.029). Consistent associations were observed at 90 and 120 days. Time-to-event analyses showed higher hazards of AF detection with early implantation (hazard ratio (HR) = 4.29 at 30 days; HR = 2.97 at 90 days; HR = 2.77 at 120 days; all p < 0.01). RMST analyses demonstrated progressively shorter time to AF diagnosis in the ICM_EARLY_ group across multiple time horizons. Results were robust across sensitivity analyses.

**Conclusion:**

Early ICM implantation after ESUS is associated with higher and faster AF detection compared with delayed implantation. When ICM monitoring is indicated, avoiding unnecessary delays may substantially enhance diagnostic yield.

## Graphical abstract

**Graphical abstract:**
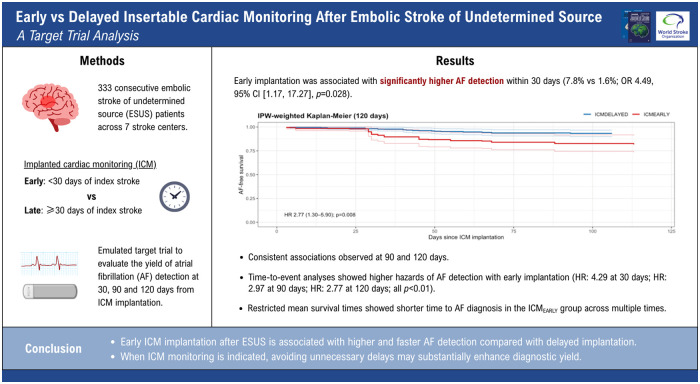

## Introduction

Ischemic stroke is a leading cause of death and disability worldwide.^1^ In up to 20–40% of patients, the cause remains elusive after standard diagnostic workup, and these cases are typically classified as cryptogenic stroke^2^ or more specifically as embolic stroke of undetermined source (ESUS).^3–5^ Atrial fibrillation (AF) is a well-established mechanism of ischemic stroke,^2,6^ and its associated risk can be markedly reduced through oral anticoagulation.^7–10^ However, initiation of anticoagulant therapy requires documented evidence of AF, while patients without such documentation are generally managed with antiplatelet agents.^11^ Because AF is frequently paroxysmal and asymptomatic, routine short-term monitoring often fails to capture arrhythmic episodes.^12^ Randomized controlled trial such as CRYSTAL-AF has shown that prolonged monitoring with implantable cardiac monitors (ICMs) substantially increases AF detection rates, identifying arrhythmias in up to 30% of ESUS patients during follow-up.^13^ Subsequent meta-analyses have further confirmed the clinical utility of ICMs, demonstrating their superiority over conventional monitoring strategies.^14,15^ Despite the widespread adoption of ICMs in post-stroke diagnostic pathways, the optimal timing of implantation after the index cerebrovascular event remains uncertain. This question is clinically relevant, as delayed AF diagnosis may postpone initiation of anticoagulation and expose patients to an increased risk of recurrent stroke. Recently, a large systematic review and meta-analysis explored the relationship between timing of ICM implantation and AF detection, suggesting that earlier implantation is associated with higher diagnostic yield and shorter time to AF diagnosis, independent of monitoring duration.^16^ However, this evidence was derived from heterogeneous study-level data and observational designs not specifically structured to address timing as a causal exposure, leaving the potential for selection bias, immortal time bias, and confounding by indication. Therefore, whether early versus delayed ICM implantation causally influences AF detection and diagnostic delay in patients with ESUS remains an open question that requires investigation within a study specifically designed to emulate a randomized comparison. In this multicenter observational cohort study, we aimed to assess the effect of early versus delayed ICM implantation on AF detection and time to diagnosis in patients with ESUS. To minimize bias inherent to observational data, we designed the study and analysis according to the principles of a hypothetical randomized controlled trial using a target trial emulation framework.^17,18^ We hypothesized that earlier ICM implantation would be associated with a higher AF detection rate and a shorter time to AF diagnosis compared with delayed implantation.

## Methods

We conducted a retrospective, multicenter, observational cohort study of patients with ESUS who underwent ICM implantation. To minimize biases inherent in observational research, we applied a target trial emulation framework,^17,18^ aiming to replicate as closely as possible the conditions of a randomized controlled trial comparing early (⩽30 days, ICM_EARLY_) versus delayed (31–365 days, ICM_DELAYED_) ICM implantation after the index event. This approach required explicitly defining eligibility criteria, exposure assignment, start of follow-up, and outcome definitions in line with a hypothetical trial protocol, thereby reducing risks of selection bias and immortal time bias. In this framework, all patients entered follow-up at the time of ICM implantation (time zero), with group allocation defined by the elapsed time since the qualifying ESUS event. We prespecified baseline covariates for confounding adjustment, mirroring the variables that would have been considered for randomization in a trial setting, and ensured complete capture of outcomes during standardized follow-up. Retrospective data were drawn from prospective, ongoing stroke registries at seven stroke centers (Supplemental Table S1), which included cases between 2021 and 2024 internationally. Anonymized data were pooled centrally. Where applicable, approval from local ethics committees was obtained. We adhered to the STROBE guidelines^19^ to ensure accurate and transparent reporting of the study findings. The analysis plan was finalized and circulated before data collection and analysis. Individual patient data from this study cannot be shared due to data protection regulations; however, aggregated data can be provided upon request.

### Patient selection

Consecutive patients captured in the prospective registries of the participating comprehensive stroke centers were screened for eligibility for the emulated target trial study analysis (Supplemental Figure S1). Eligible patients were 18 years of age or older and had received a diagnosis of ESUS stroke or TIA. Ischemic stroke was defined as a new neurological deficit with a corresponding acute ischemic lesion on brain NCCT or MRI, including cases with symptom resolution within 24 h (TIAs). ESUS was defined according to the criteria proposed by the Cryptogenic Stroke/ESUS International Working Group, as follows: (1) ischemic stroke detected by magnetic resonance or computed tomography imaging that is not lacunar, (2) absence of extracranial stenosis causing ⩾50% luminal stenosis in arteries supplying the area of ischemia detected with a magnetic resonance angiography or computed tomography angiogram, (3) no major-risk cardioembolic source, and (4) no other specific cause of stroke identified (e.g. arteritis, dissection, vasospasm, drug misuse).^4^ To ensure methodological consistency across participating centers during the study period (2021–2024), the original 2014 operational ESUS definition was applied uniformly to all patients.^4^ Imaging interpretation and ESUS classification were performed locally at each participating center by experienced stroke neurologists and neuroradiologists in accordance with predefined diagnostic criteria and standardized evaluation pathways. No central imaging adjudication committee was employed. All participating centers are certified comprehensive stroke centers with established and protocolized diagnostic pathways for ESUS evaluation. In some patients, extracranial carotid ultrasound was combined with CTA or MRA of the intracranial circulation whereas in others CTA or MRA was performed for both extracranial and intracranial vessels. For this analysis, we excluded patients with life expectancy less than 6 months, prosthetic mechanical valve, pacemaker, hepatic disease associated with coagulopathy (prothrombin time prolonged beyond the normal range), and clinically relevant bleeding risk including cirrhotic patients with Child Pugh B and C and estimated glomerular filtration rate (eGFR) < 15 mL/min/1.73 m^2^ as well as those discharged on oral anticoagulants after the index ESUS event, or who developed AF or experienced any recurrent ischemic or cardiovascular event between the index event and ICM implantation (study flow chart, Supplemental Figure S1). Twelve-lead electrocardiography, transthoracic or transoesophageal echocardiography, and cardiac monitoring for at least 24 h were performed before determining the indication for ICM implantation. After written informed consent, the patients underwent ICM implantation under local anesthesia. Patients underwent ICM with Reveal LINQ, Medtronic Inc., Minneapolis, MN, USA. Outcome assessment was carried out during scheduled outpatient visits and/or through contact with the patient, their relatives, or the patient’s primary physician. At each evaluation, all available clinical documentation was reviewed, including hospital discharge summaries, correspondence from primary care physicians, and reports of investigations performed in the outpatient setting. The timing of ICM implantation was determined at the discretion of the treating physician. AF was defined as an episode of irregular heart rhythm without detectable P waves lasting more than 30 s,^20,21^ and consistent with prior studies in this field.^13^ Time variables were collected prospectively and included day of stroke onset, ICM implantation, and first AF detection. The timing of ICM implantation was determined at the discretion of the treating physician. Follow-up information was obtained by systematic review of ICM downloads, hospital records, and outpatient clinic visits at each site.

### Definition of variables

All variables were prospectively collected at baseline according to standardized definitions (see supplemental material).

### Outcome measures

The prespecified primary outcome was AF detection within 30 days of ICM implantation. AF detection was assessed using two complementary measures: (1) cumulative incidence, defined as the proportion of patients with at least one AF episode detected within the specified time window, and (2) AF detection rate, expressed as the number of AF events per person-time at risk. Time at risk accrued from the date of ICM implantation until AF detection or censoring at the end of the corresponding time horizon. Secondary outcomes included AF detection within 90 and 120 days from ICM implantation and evaluated using the same outcome definitions as the primary analysis. For each time horizon, cumulative incidence and AF detection rates were estimated separately for the early (ICMEARLY, ⩽30 days from index event) and delayed (ICMDELAYED, 31–365 days) implantation strategies. Effect estimates were derived using inverse probability weighting to account for baseline confounding. Differences in cumulative incidence were quantified using weighted odds ratios (ORs), while differences in AF detection rates were quantified using weighted incidence rate ratios (IRRs). All outcomes were analyzed according to the prespecified target trial emulation framework, with follow-up starting at the time of ICM implantation and outcomes assessed within fixed post-implantation time windows.

### Statistical analysis

Analyses were conducted according to a prespecified target trial emulation framework. Baseline characteristics were summarized using counts and percentages for categorical variables and medians with interquartile ranges (IQRs) for continuous variables. Between-group differences were assessed using the chi-square or Fisher’s exact tests for categorical variables and Wilcoxon rank-sum tests for continuous variables, as appropriate. To estimate the causal effect of early versus delayed ICM implantation on AF detection, inverse probability weighting (IPW) was used to adjust for baseline confounding. Propensity scores for early implantation were estimated using a logistic regression model including prespecified baseline covariates selected a priori based on clinical relevance. Stabilized weights were applied to create a pseudo-population in which baseline covariates were balanced between exposure groups. Covariate balance before and after weighting was assessed using standardized mean differences. Variables included in the propensity score model and key exposure and outcome-related variables were mandatory registry fields and complete for all included patients; therefore, no imputation was required for the primary analyses. For the primary outcome (AF detection within 30 days from implantation) and secondary outcomes (AF detection within 90 and 120 days), cumulative incidence was compared between groups using weighted logistic regression models, with results reported as ORs and 95% confidence intervals (CIs). AF detection rates per person-time were compared using weighted Poisson regression models with a log link and offset for person-time at risk, and results were reported as IRRs with 95% CIs. Robust (sandwich) variance estimators were used to account for weighting. Time-to-event analyses were performed using weighted Cox proportional hazards models, with time zero defined as the date of ICM implantation and follow-up censored at the end of the corresponding time horizon. Proportional hazards assumptions were assessed using Schoenfeld residuals. To complement hazard-based estimates and provide an absolute measure of diagnostic delay, restricted mean survival time (RMST) was calculated at prespecified time horizons, and between-group differences in RMST were reported with 95% CIs. Several sensitivity analyses were performed to assess the robustness of the primary findings. First, all weighted outcome models were re-estimated using robust sandwich variance estimators clustered at the recruiting center level to account for within-center correlation and potential center-specific practice patterns. Second, time-to-event analyses were repeated using Fine–Gray subdistribution hazard models to account for competing risks, yielding consistent effect estimates across time horizons. Third, RMST differences were estimated at multiple prespecified time horizons to evaluate the robustness of conclusions regarding earlier AF detection independently of proportional hazards assumptions. Across all sensitivity analyses, the direction and magnitude of effect estimates were consistent with the main analyses. All hypothesis tests were two-sided, and a p value < 0.05 was considered statistically significant. Analyses were performed using R (version 2024).

### Sample size calculation

Because this study was designed as a retrospective target trial emulation including all consecutive eligible patients, no formal a priori sample size calculation was performed. The sample size was, therefore, determined by the number of patients meeting prespecified eligibility criteria across participating centers. As a feasibility and precision assessment, we considered whether the observed number of AF events was sufficient to support the prespecified primary comparison between early and delayed ICM implantation. The final analytic cohort included 333 patients, of whom 90 (27%) underwent early ICM implantation, and 243 (73%) underwent delayed implantation. Although the number of AF events within the earliest time windows was limited, effect estimates were consistently robust across complementary analytic approaches, including cumulative incidence, incidence rates, time-to-event analyses, and restricted mean survival time, supporting the adequacy of the available sample to address the primary study hypothesis. These estimated event rates are grounded in prior literature.^13,22,23^

## Results

Overall, the target trial cohort included 333 ESUS patients; the median age was 67 (IQR = 57–74) years, 62.8% were male (Table 1, Supplemental Figure S1). Of these, 90 (27%) received ICM ⩽ 30 days since the index event (ICM_EARLY_) while 243 (73%) received ICM between 31 and 365 days from the index event (ICM_DELAYED_). Baseline vascular risk factors did not differ significantly according to timing of ICM implantation. A numerically higher proportion of patients in the ICM_EARLY_ group had a history of prior TIA or ischemic stroke (32.2% vs 21.8%; p = 0.050) and were receiving antiplatelet therapy before the index event (38.9% vs 26.7%; p = 0.032) compared with ICM_DELAYED_ group. Cardiac structural abnormalities and rhythm-related findings were comparable between groups, including abnormal left atrial parameters, valvular disease, presence of patent foramen ovale, supraventricular tachycardia, and atrial ectopic activity. Stroke severity on admission was low overall, with a median NIHSS score of 2 (IQR = 0–5), although patients in the ICM_EARLY_ group had slightly higher NIHSS (National Institute of Health Stroke Scale) scores at presentation (median = 3 vs 2; p = 0.014). The number of infarcts on brain imaging and CHA_2_DS_2_-VASc scores were similar between groups. Laboratory biomarkers, including B-type natriuretic peptide (BNP), troponin, and TSH levels, did not differ significantly between early and delayed implantation groups. Median duration of ICM monitoring exceeded 2 years and was comparable between groups.

**Table 1.** Baseline characteristics.

	Overall (N = 333)	ICM_EARLY_ (N = 90)	ICM_DELAYED_ (N = 243)	p
Age, median (IQR), y	67 (57–74)	66.5 (57–74)	67 (56.8–74)	0.887
Sex, male, No. (%)	209 (62.8)	60 (66.7)	149 (61.3)	0.370
Type of ESUS, No. (%)				0.254
TIA	45 (13.5)	9 (10)	36 (14.8)	
IS	288 (86.5)	81 (90.0)	207 (85.2)	
**Risk factors**				
Hypertension, No. (%)	191 (57.4)	55 (61.1)	136 (56.0)	0.399
Diabetes, No. (%)	67 (20.1)	44 (48.9)	93 (38.3)	0.115
Dyslipidemia, No. (%)	137 (41.1)	96 (33)	83 (42)	0.080
Coronary artery disease, No. (%)	46 (13.8)	16 (17.8)	30 (12.3)	0.202
Heart failure, No. (%)	18 (5.4)	5 (5.6)	13 (5.3)	1.000
Previous TIA/Ischemic stroke No. (%)	82 (24.6)	29 (32.2)	53 (21.8)	0.050
Alcohol abuse, No. (%)	10 (3.0)	0 (0.0)	10 (4.1)	0.344
Current/Former smoker, No. (%)	133 (39.9)	43 (47.8)	90 (37.0)	0.076
Malignancy, No. (%)	31 (9.3)	8 (8.9)	23 (9.5)	0.872
**Cardiac biomarkers**				
Abnormal LA, No. (%)	122 (36.6)	30 (33.3)	92 (37.9)	0.446
Abnormal mitral valve, No. (%)	79 (23.7)	25 (27.8)	54 (22.2)	0.290
Abnormal aortic valve, No. (%)	36 (10.8)	10 (11.1)	26 (10.7)	0.914
PFO, No. (%)	75 (22.5)	25 (27.8)	50 (20.6)	0.162
Presence of SVT, No. (%)	56 (16.8)	14 (15.6)	42 (17.3)	0.708
Presence of atrial ectopics, No. (%)	130 (39.0)	30 (33.3)	100 (41.2)	0.194
**TIA/Stroke characteristics**				
NIHSS on admission, median (IQR)	2 (0–5)	3 (0.2–7)	2 (0–4)	0.014
No. of infarcts, median (IQR)	2 (1–3)	2 (1–3)	1 (1–3)	0.645
CHA_2_DS_2_-VASc Score, median (IQR)	4 (3–5)	4 (3–5)	4 (3–5)	0.273
Previous treatment with antiplatelet agent, No. (%)	100 (30.0)	35 (38.9)	65 (26.7)	0.032
**Blood sample analysis**				
BNP, median, pg/mL (IQR)	85.5 (58–323.2)	120.5 (57.2–446.2)	79.5 (58.2–235.8)	0.642
Troponin, median, ng/L (IQR)	5 (3–12.3)	4.2 (3–14.2)	5 (3–12)	0.907
TSH, median, mIU/L (IQR)	1.4 (1.1–2.3)	1.3 (1–2.3)	1.5 (1.1–2.4)	0.608
ICM monitoring, days, median (IQR)	1031 (739.5–1402)	928 (722–1495.2)	1055 (766–1378)	0.706

AF: atrial fibrillation; BNP: B-type natriuretic peptide; ESUS: embolic stroke of undetermined source; ICM: implantable cardiac monitor; ICMEARLY: ICM implantation ⩽30 days after index event; ICMDELAYED: ICM implantation between 31 and 365 days after index event; IQR: interquartile range; IS: ischemic stroke; LA: left atrium; NIHSS: National Institutes of Health Stroke Scale; No.: number; PFO: patent foramen ovale; SVT: supraventricular tachycardia; TIA: transient ischemic attack; TSH: thyroid-stimulating hormone.

Covariate balance between the ICM_EARLY_ and ICM_DELAYED_ groups before and after inverse probability weighting is shown in Supplemental Table S2 (Supplemental Figures S2 and S3). Before weighting, several baseline characteristics exhibited meaningful imbalance between groups, including stroke severity on admission (NIHSS: SMD = 0.28), CHA_2_DS_2_-VASc score (SMD = 0.15), BNP levels (SMD = 0.23), and troponin levels (SMD = 0.18). Additional, though more modest, imbalances were observed for prior TIA or ischemic stroke, atrial ectopic activity, and structural cardiac abnormalities. After application of stabilized IPW, covariate balance substantially improved across nearly all measured variables (Supplemental Table S2, Supplemental Figures S2 and S3). Absolute SMDs were reduced to below the prespecified threshold of 0.10 for the majority of covariates, including age, sex, ESUS subtype, vascular risk factors, cardiac structural parameters, NIHSS score, CHA_2_DS_2_-VASc score, BNP, and number of infarcts. Residual imbalance was minimal, with the largest post-weighting SMD observed for troponin (SMD = 0.12) and TSH (SMD = 0.06), both remaining close to the threshold for acceptable balance.

### Study outcomes

**Figure 1.**
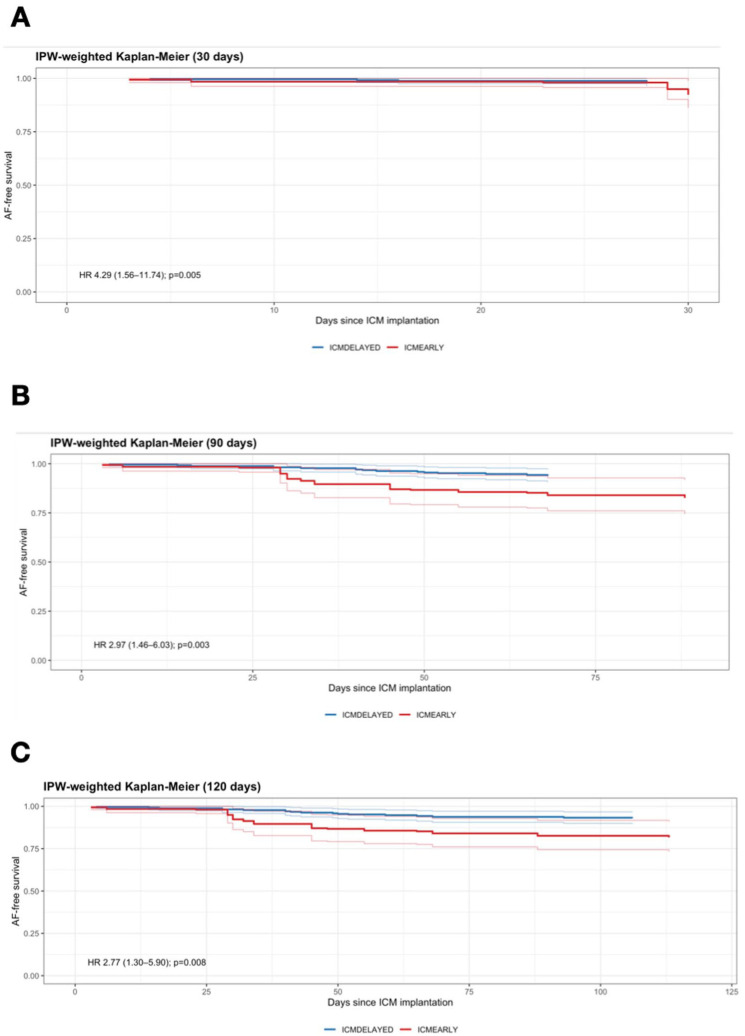
**IPW-weighted Kaplan–Meier curves for time to atrial fibrillation detection.** IPW-weighted Kaplan–Meier curves showing atrial fibrillation (AF)–free survival according to timing of implantable cardiac monitor (ICM) implantation. Time zero was defined as the date of ICM implantation. Early implantation (ICMEARLY) was defined as ICM implantation ⩽30 days after the index ESUS event and delayed implantation (ICMDELAYED) as implantation between 31 and 365 days. **Panel A** shows AF-free survival within 30 days after implantation. **Panel B** shows AF-free survival within 90 days after implantation. **Panel C** shows AF-free survival within 120 days after implantation. Curves were estimated using inverse probability weighting (IPW) to adjust for baseline confounding. Shaded areas represent 95% confidence intervals. Hazard ratios (HRs) and corresponding 95% confidence intervals were estimated using IPW-weighted Cox proportional hazards models with robust variance estimation. AF = atrial fibrillation; ICM = implantable cardiac monitor; IPW = inverse probability weighting.

Primary and secondary outcomes of AF detection according to the timing of ICM implantation are summarized in Table 2. For the primary outcome, AF within 30 days after implantation was detected in 7 of 90 patients (7.8%) in the ICM_EARLY_ group and in 4 of 243 patients (1.6%) in the ICM_DELAYED_ group. In the IPW-weighted analysis, early implantation was associated with higher odds of AF detection (OR = 4.49, 95% CI = 1.17–17.27; p = 0.028) and a higher AF detection rate per person-time (IRR = 4.26, 95% CI = 1.16–15.60; p = 0.029). Consistent results were observed in time-to-event analyses. Early ICM implantation was associated with a significantly higher hazard of AF detection within 30 days compared with delayed implantation (hazard ratio (HR) = 4.29, 95% CI = 1.56–11.74; p = 0.005) (Figure 1A). For secondary outcomes, the association remained consistent at 90 and 120 days. At 90 days, cumulative AF detection was 17.8% versus 5.8% (OR = 2.91, 95% CI = 1.28–6.61; p = 0.011), with a higher detection rate in the ICM_EARLY_ group (IRR = 2.98, 95% CI = 1.38–6.44; p = 0.005). Time-to-event analysis confirmed a higher hazard of AF detection associated with early implantation (HR = 2.97, 95% CI = 1.46–6.03; p = 0.003) (Figure 1B). Similar findings were observed at 120 days (OR = 2.77, 95% CI = 1.26–6.07; p = 0.011; IRR = 2.81, 95% CI = 1.34–5.89; p = 0.006). This was paralleled by a significantly increased hazard of AF detection in the early implantation group (HR = 2.77, 95% CI = 1.30–5.90; p = 0.008) (Figure 1C).

**Table 2.** Primary and secondary outcomes of AF detection according to timing of ICM implantation.

	Time horizon, (days)	ICM_EARLY_ (N = 90)	ICM_DELAYED_ (N = 243)	IPW-weighted effect estimate (95% CI)	p
**Primary outcomes—AF detection within 30** **days**					
Cumulative incidence, No. (%)	30	7 (7.8)	4 (1.6)	OR = 4.49 (1.17–17.27)	0.028
AF detection rate (per person-time)	30	–	–	IRR = 4.26 (1.16–15.60)	0.029
**Secondary outcomes—AF detection within 90 and 120** **days**					
Cumulative incidence, No. (%)	90	16 (17.8)	14 (5.8)	OR = 2.91 (1.28–6.61)	0.011
AF detection rate (per person-time)	90	–	–	IRR = 2.98 (1.38–6.44)	0.005
Cumulative incidence, No. (%)	120	17 (18.9)	16 (6.6)	OR = 2.77 (1.26–6.07)	0.011
AF detection rate (per person-time)	120	–	–	IRR = 2.81 (1.34–5.89)	0.006

AF: atrial fibrillation; ICM: implantable cardiac monitor; ICMEARLY: ICM implantation ⩽30 days after index event; ICMDELAYED: ICM implantation between 31 and 365 days after index event; CI: confidence interval.

### Exploratory analysis

**Figure 2.**
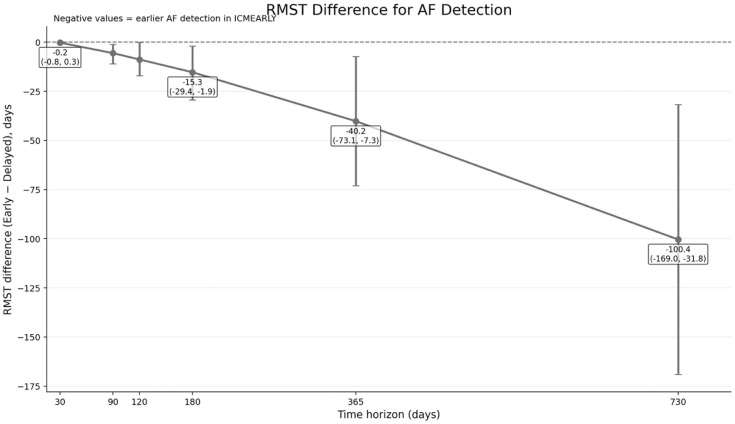
**Restricted mean survival time (RMST) difference for time to atrial fibrillation detection.** Restricted mean survival time (RMST) difference for time to first atrial fibrillation (AF) detection comparing early versus delayed implantable cardiac monitor (ICM) implantation across prespecified time horizons. RMST differences are expressed as days and calculated as RMST in the ICMEARLY group minus RMST in the ICMDELAYED group. Negative values, therefore, indicate earlier AF detection in the ICMEARLY group. Points represent estimated RMST differences at each time horizon (30, 90, 120, 180, 365, and 730 days), with vertical bars indicating 95% confidence intervals derived from IPW-based RMST regression models. The dashed horizontal line at zero represents no difference between groups. Time zero was defined as the date of ICM implantation. AF = atrial fibrillation; ICM = implantable cardiac monitor; IPW = inverse probability weighting.

Table 3 and Figure 2 report the RMST for time to first AF detection across prespecified time horizons. At 30 days, no significant difference in RMST was observed between the ICM_EARLY_ and ICM_DELAYED_ groups (RMST difference = −0.23 days; 95% CI = −0.85 to 0.28; p = 0.466). From 90 days onward, early ICM implantation was consistently associated with shorter time to AF detection. At 90 days, the RMST was 81.3 days in the ICM_EARLY_ group versus 86.9 days in the ICM_DELAYED_ group, corresponding to a mean difference of −5.59 days (95% CI = −11.0 to −1.07; p = 0.026). This difference increased progressively at longer horizons, reaching −8.82 days at 120 days (p = 0.021), −15.3 days at 180 days (p = 0.025), −40.2 days at 365 days (p = 0.011), and −100.4 days at 730 days (p = 0.004).

**Table 3.** Restricted mean survival time (RMST) for time to AF detection.

Time horizon (days)	RMST ICM_EARLY_	RMST ICM_DELAYED_	RMST difference (early–delayed), days (95% CI)	p
30	29.5	29.8	−0.23 (−0.85 to 0.28)	0.466
90	81.3	86.9	−5.59 (−11.0 to −1.07)	0.026
120	106.0	115.0	−8.82 (−17.1 to −2.06)	0.021
180	154.7	170.0	−15.3 (−29.4 to −1.9)	0.025
365	294.8	335.0	−40.2 (−73.1 to −7.3)	0.011
730	629.6	730.0	−100.4 (−169.0 to −31.8)	0.004

Restricted mean survival time (RMST) represents the mean time to first atrial fibrillation (AF) detection up to the specified time horizon. RMST differences are expressed in days and were calculated as RMST in the ICMEARLY group minus RMST in the ICMDELAYED group; negative values indicate earlier AF detection in the ICMEARLY group. *p* values were derived from RMST regression models.

### Sensitivity analyses

Sensitivity analyses yielded results consistent with the primary analyses (Supplemental Table S3). Across all time horizons, early ICM implantation remained associated with a higher probability and faster detection of AF compared with delayed implantation. At 30 days, the association persisted across all modeling approaches, including IPW-weighted logistic regression (OR = 4.18, 95% CI = 1.09–16.01), Poisson regression (IRR = 3.96, 95% CI = 1.42–11.01), Cox proportional hazards models (HR = 3.92, 95% CI = 1.08–14.21), and competing-risk Fine–Gray models accounting for death and recurrent non-AF cardiovascular events (sHR = 3.88, 95% CI = 1.07–14.05). Similar concordant estimates were observed at 90 and 120 days, with effect sizes remaining directionally consistent across logistic, rate-based, time-to-event, and competing-risk frameworks. Restricted mean survival time analyses supported these findings, demonstrating a shorter mean time to AF detection in the ICM_EARLY_ group at longer time horizons, although RMST differences did not reach statistical significance at all prespecified cutoffs.

## Discussion

In this multicenter observational study emulating a target trial, we found that early implantation of an ICM after ESUS was consistently associated with higher AF detection and a shorter time to diagnosis compared with delayed implantation. Early ICM implantation was associated with a markedly higher cumulative incidence of AF across all prespecified time horizons, with the largest absolute and relative differences observed within the first 30 days following device insertion. These early differences persisted over time, remaining evident at 90 and 120 days, indicating that the benefit of earlier monitoring is not limited to an initial detection spike but reflects a sustained increase in diagnostic yield. Beyond cumulative incidence, early implantation was also associated with significantly higher AF detection rates per person-time, demonstrating that the observed effect was not solely driven by longer observation windows but reflected a higher intensity of AF capture over time. Importantly, complementary restricted mean survival time analyses provided an absolute and clinically intuitive measure of diagnostic delay. These analyses showed that delayed implantation was associated with progressively longer delays to AF diagnosis, with differences increasing steadily over longer follow-up horizons. By 1 year and beyond, delayed implantation translated into several weeks to months of additional AF-free time compared with early implantation, underscoring the clinical relevance of earlier monitoring strategies. Crucially, the direction and magnitude of these associations were robust across a range of analytical approaches and sensitivity analyses. Together, these converging lines of evidence strongly support a causal interpretation whereby earlier ICM implantation after ESUS leads to faster and more frequent detection of AF.

Our results align with and expand upon prior evidence supporting the role of prolonged cardiac monitoring in ESUS patients suggesting that AF may be particularly active or detectable in the acute phase following a cerebrovascular event.^24^ The concept of “Atrial Fibrillation Detected After Stroke” (AFDAS) has gained traction as a distinct clinical entity characterized by a temporally heightened vulnerability to arrhythmia in the days to weeks after stroke or TIA.^25,26^

The CRYSTAL-AF study and subsequent meta-analyses confirmed the incremental value of ICMs in ESUS patients, with pooled detection rates approaching 30% over follow-up.^13,15^ However, those studies largely focused on device efficacy versus control and did not systematically evaluate the timing of implantation. Then, a recent meta-analysis by Sposato et al. demonstrated that initiating continuous cardiac monitoring earlier after stroke increased the likelihood of AF detection more than fivefold. In line with this, prior studies have shown that the majority of AF cases are identified very early, with over 70% detected within the first 72 h of admission. Importantly, however, this meta-analysis did not specifically address the impact of ICM implantation timing, and its conclusions regarding early monitoring are primarily based on non-ICM modalities. Therefore, our analysis addresses this critical knowledge gap, suggesting that not only whether but also when an ICM is implanted influences its diagnostic performance. From a clinical standpoint, earlier implantation translates into earlier identification of occult AF, potentially allowing earlier initiation of anticoagulation therapy.

The clinical implications of our findings are relevant for clinical practice. While current guidelines endorse prolonged cardiac monitoring in ESUS, they provide no recommendations on when to initiate ICM implantation.^27^ In everyday practice, as reflected in our multicenter cohort, implantation timing is largely influenced by physician judgment, patient characteristics, and local organizational factors such as waiting lists and pathway structure. Our results suggest that a strategy of early ICM implantation may enhance AF detection and shorten diagnostic delay, particularly in the first months after implantation, potentially allowing earlier initiation of secondary prevention in patients at higher embolic risk. At the same time, earlier implantation has implications for resource use, costs, and patient selection, and it remains uncertain whether earlier AF detection translates into improved long-term clinical outcomes. Further prospective studies are needed to define the optimal balance between diagnostic yield, cost-effectiveness, and clinical benefit.

Our study has several strengths. First, we applied a target trial emulation framework, explicitly defining eligibility, exposure assignment, and outcomes to minimize biases such as immortal time bias. Second, rigorous propensity score weighting achieved excellent covariate balance, approximating the conditions of a randomized trial. Third, the use of multiple complementary statistical approaches, including RMST, strengthened the robustness of findings. Finally, the multicenter design, including diverse international stroke centers, might enhance generalizability. Nevertheless, some limitations merit consideration. As an observational study, residual confounding cannot be fully excluded despite weighting and adjustment. Implantation timing was determined by treating physicians, and in some centers, early implantation was systematic, whereas others prioritized extended Holter monitoring. Logistical factors, including waiting lists, also influenced implantation decisions. However, potential confounding by indication was addressed through a prespecified target trial emulation framework and inverse probability weighting, achieving substantial post-weighting covariate balance across clinically relevant variables. While unmeasured confounding cannot be entirely excluded, the consistency of findings across multiple complementary analytic approaches supports the robustness of the observed association. Since inclusion was based on the original 2014 ESUS criteria,^4^ our cohort reflects standardized real-world diagnostic practice during the study period. While recent updates to the ESUS construct^28^ have proposed refinement of certain subgroups—particularly reclassification of younger patients with high-risk PFO features—reassessment of our cases confirmed alignment with these contemporary recommendations. No patients meeting criteria for high-risk PFO-associated stroke were included under the ESUS classification. Finally, imaging interpretation and ESUS classification were performed locally without central adjudication. Although all participating centers are certified comprehensive stroke centers with standardized diagnostic pathways, inter-center variability in imaging interpretation cannot be entirely excluded.

In conclusion, this multicenter target trial emulation demonstrates that early ICM implantation after ESUS is associated with significantly higher AF detection and shorter time to diagnosis compared with delayed implantation. These findings suggest that earlier deployment of ICMs could improve the efficiency of arrhythmia detection and facilitate the timely initiation of anticoagulation. While confirmatory randomized trials are warranted, our results support consideration of earlier ICM implantation as part of optimized secondary prevention strategies in patients with ESUS.

## Supplemental Material

sj-docx-1-wso-10.1177_17474930261438742 – Supplemental material for Timing of insertable cardiac monitor implantation after embolic stroke of undetermined source and its impact on atrial fibrillation detection: A target trial emulation analysis Supplemental material, sj-docx-1-wso-10.1177_17474930261438742 for Timing of insertable cardiac monitor implantation after embolic stroke of undetermined source and its impact on atrial fibrillation detection: A target trial emulation analysis by Lucio D’Anna, Fionn Maguidhir, Robert Simister, Arvind Chandratheva, Gaurav Desai, Maryam Haneef, Anne Idian, Francesco Favruzzo, Alessandra Pes, Claudio Baracchini, Diletta Rosin, Mariarosaria Valente, Gian Luigi Gigli, Liqun Zhang, Nathan Leung Mres, Manav Sohal, Simona Sacco, Raffaele Ornello, Federico De Santis, Ubaldo Coppola, Gabriele Prandin, Selina Edwards, Ceylan Safak, Roberto Avila, Joan Cruz, Ashley Laurie, Michele Romoli, Valentina Tudisco, Federica Nicoletta Sepe, Jianqun Guan, Asha Barnard, Lydia Jeffrey, Jake Dagan, Tsering Dolkar, Jonathan Hayton, Soma Banerjee, Matteo Foschi, Giovanni Merlino and Phang Boon Lim in International Journal of Stroke

sj-docx-2-wso-10.1177_17474930261438742 – Supplemental material for Timing of insertable cardiac monitor implantation after embolic stroke of undetermined source and its impact on atrial fibrillation detection: A target trial emulation analysis Supplemental material, sj-docx-2-wso-10.1177_17474930261438742 for Timing of insertable cardiac monitor implantation after embolic stroke of undetermined source and its impact on atrial fibrillation detection: A target trial emulation analysis by Lucio D’Anna, Fionn Maguidhir, Robert Simister, Arvind Chandratheva, Gaurav Desai, Maryam Haneef, Anne Idian, Francesco Favruzzo, Alessandra Pes, Claudio Baracchini, Diletta Rosin, Mariarosaria Valente, Gian Luigi Gigli, Liqun Zhang, Nathan Leung Mres, Manav Sohal, Simona Sacco, Raffaele Ornello, Federico De Santis, Ubaldo Coppola, Gabriele Prandin, Selina Edwards, Ceylan Safak, Roberto Avila, Joan Cruz, Ashley Laurie, Michele Romoli, Valentina Tudisco, Federica Nicoletta Sepe, Jianqun Guan, Asha Barnard, Lydia Jeffrey, Jake Dagan, Tsering Dolkar, Jonathan Hayton, Soma Banerjee, Matteo Foschi, Giovanni Merlino and Phang Boon Lim in International Journal of Stroke
