# Supplementary material for: Timing of insertable cardiac monitor implantation after embolic stroke of undetermined source and its impact on atrial fibrillation detection: A target trial emulation analysis
Source: Int J Stroke. 2026 Mar 24;21(7):968–78. doi: 10.1177/17474930261438742 (PMC13392176; doi:10.1177/17474930261438742)
Supplement: sj-docx-1-wso-10.1177_17474930261438742 – Supplemental material for Timing of insertable cardiac monitor implantation after embolic stroke of undetermined source and its impact on atrial fibrillation detection: A target trial emulation analysis [file sj-docx-1-wso-10.1177_17474930261438742.docx]

**Supplemental material**

*Data collection*

Baseline demographic, clinical, cardiac, and laboratory variables were defined as follows:

Age was recorded in years at the time of the index event. Sex was categorized as male or female. Type of ESUS distinguished ischemic stroke (IS) from transient ischemic attack (TIA) according to standard clinical and imaging criteria. Hypertension, diabetes mellitus, dyslipidaemia, coronary artery disease, and heart failure were defined according to prior clinical diagnosis documented in medical records or ongoing treatment with corresponding medications. Previous TIA/ischemic stroke indicated any prior cerebrovascular event preceding the index ESUS. Alcohol abuse referred to chronic or hazardous alcohol consumption documented in clinical notes, and current/former smoker included both active and past tobacco users. Malignancy referred to any active or previous cancer diagnosis (excluding non-melanoma skin cancer). Cardiac parameters were obtained from transthoracic or transoesophageal echocardiography and electrocardiographic monitoring. Abnormal left atrium (LA) indicated atrial enlargement or structural abnormality. Abnormal mitral and aortic valve referred to the presence of any structural or functional valve pathology (e.g., stenosis or regurgitation of at least mild degree). Patent foramen ovale (PFO) was diagnosed by echocardiographic evidence of right-to-left shunt at rest or after Valsalva maneuver. Presence of supraventricular tachycardia (SVT) or atrial ectopics was based on ECG or Holter findings. Abnormal PR interval referred to any deviation from the normal range (120–200 ms). NIHSS on admission indicated stroke severity at presentation, measured by the National Institutes of Health Stroke Scale. Number of infarcts represented the total number of ischemic lesions identified on brain MRI or CT. CHA₂DS₂-VASc score was calculated according to established criteria based on age, sex, and vascular risk factors. Previous treatment with antiplatelet agent indicated ongoing or recent antiplatelet therapy at the time of the index event.

BNP (B-type natriuretic peptide), troponin, and TSH (thyroid-stimulating hormone) levels were collected from admission laboratory data. Holter duration referred to the total hours or days of pre-implant ECG monitoring before ICM implantation. ICM monitoring duration was defined as the total number of days between ICM implantation and the last available follow-up or explantation. All definitions were standardized across centres through a shared data dictionary and verified locally before data pooling.
