# Supplementary material for: Timing of insertable cardiac monitor implantation after embolic stroke of undetermined source and its impact on atrial fibrillation detection: A target trial emulation analysis
Source: Int J Stroke. 2026 Mar 24;21(7):968–78. doi: 10.1177/17474930261438742 (PMC13392176; doi:10.1177/17474930261438742)

**Table S1. Site contribution.**

| **Site** | **Target trial patients** | **Standard Operating Procedure (SOP)** |
| --- | --- | --- |
| **Imperial College London** | 239 | Yes |
| **Udine** | 45 | Yes |
| **Cesena** | 13 | Yes |
| **L’Aquila** | 14 | Yes |
| **Padua** | 60 | Yes |
| **St George London** | 37 | Yes |
| **UCL London** | 76 | Yes |

**Table S2. Covariate balance before and after inverse probability weighting (IPW) between the two groups**

|  | **SMD unweighted** | **SMD weighted** |
| --- | --- | --- |
| Age | 0.0231 | 0.0204 |
| Sex, Male | 0.0535 | 0.0054 |
| Type of ESUS, TIA or IS | 0.0481 | 0.0095 |
| Hypertension | 0.0514 | 0.0001 |
| Diabetes | 0.0288 | 0.0033 |
| Coronary artery disease | 0.0543 | 0.0169 |
| Heart failure | 0.0021 | 0.0000 |
| Previous TIA/Ischemic stroke | 0.1041 | 0.0382 |
| Abnormal LA | 0.0453 | 0.0140 |
| Abnormal Mitral valve | 0.0556 | 0.0039 |
| Abnormal Aortic valve | 0.0041 | 0.0171 |
| Presence of SVT | 0.0173 | 0.0201 |
| Presence of atrial ectopics | 0.0782 | 0.0307 |
| NIHSS on admission | 0.2803 | 0.0069 |
| N of infarcts | 0.0494 | 0.0021 |
| CHA_2_DS_2_-VASc Score | 0.1519 | 0.0212 |
| BNP, median | 0.2260 | 0.0026 |
| Troponin | 0.1779 | 0.1159 |
| TSH, median | 0.1527 | 0.0571 |

*Legend: BNP, B-type natriuretic peptide; ESUS, embolic stroke of undetermined source; ICM, implantable cardiac monitor; IS, ischemic stroke; LA, left atrium; NIHSS, National Institutes of Health Stroke Scale; No., number,; SVT, supraventricular tachycardia; TIA, transient ischemic attack; TSH, thyroid-stimulating hormone. Standardized mean differences (SMDs) were calculated to assess covariate balance before (unweighted) and after (weighted) application of IPW. An absolute SMD < 0.10 was considered indicative of adequate balance.*

**Table S3. Sensitivity analyses including competing-risk modelling.**

| **Time horizon (days)** | **ICM_EARLY_ (n=90)** | **ICM_DELAYED_ (N=243)** | **Effect Estimate (95% CI)** | **P value** | **Analysis** |
| --- | --- | --- | --- | --- | --- |
| 30 | 7/90 (7.8%) | 4/423 (1.6%) | OR 4.18 (1.09-16.01) | 0.037 | IPW logistic, centre clustered |
|  |  |  | IRR 3.96 (1.42-11.01) | 0.037 | IPW Poisson, centre clustered |
|  |  |  | HR 3.92 (1.08-14.21) | 0.038 | IPW Cox, centre clustered |
|  |  |  | sHR 3.88 (1.07-14.05) | 0.039 | IPW Fine-Gray, centre clustered |
|  |  |  | RMST diff -2.7 days (-7.7 to 2.3) | 0.277 | IPW RMST |
| 90 | 16/90 (17.8%) | 14/243 (5.8%) | OR 2.98 (1.31-6.74) | 0.009 | IPW logistic, centre clustered |
|  |  |  | IRR 2.81 (1.29-6.10) | 0.009 | IPW Poisson, centre clustered |
|  |  |  | HR 2.80 (1.31-5.99) | 0.008 | IPW Cox, centre clustered |
|  |  |  | sHR 2.77 (1.30-5.92) | 0.008 | IPW Fine-Gray, centre clustered |
|  |  |  | RMST diff -8.0 days (-16.9 to 0.9) | 0.090 | IPW RMST |
| 120 | 17/90 (18.9%) | 16/243 (6.6%) | OR 2.84 (1.30-6.20) | 0.009 | IPW logistic, centre clustered |
|  |  |  | IRR 2.70 (1.18-6.16) | 0.009 | IPW Poisson, centre clustered |
|  |  |  | HR 2.67 (1.26-5.63) | 0.008 | IPW Cox, centre clustered |
|  |  |  | sHR 2.63 (1.25-5.53) | 0.010 | IPW Fine-Gray, centre clustered |
|  |  |  | RMST diff -10.9 days (-23.1 to 1.3) | 0.091 | IPW RMST |

*Inverse probability weighting (IPW) was applied to account for baseline confounding between treatment groups, using stabilized weights derived from the propensity score. Robust variance estimators clustered by study centre were used in all models. Odds ratios (ORs) were estimated using weighted logistic regression, incidence rate ratios (IRRs) using weighted Poisson regression with log person-time as offset, and hazard ratios (HRs) using weighted Cox proportional hazards models. Competing-risk analyses were performed using weighted Fine–Gray subdistribution hazard models, treating death or recurrent non-AF cardiovascular events as competing events; results are reported as subdistribution hazard ratios (sHRs). Restricted mean survival time (RMST) differences were estimated using weighted pseudo-value regression models up to the prespecified time horizon; RMST differences are expressed as ICMEARLY minus ICMDELAYED, with negative values indicating earlier AF detection in the ICMEARLY group. AF indicates atrial fibrillation; CI, confidence interval; ICM, implantable cardiac monitor; ICMEARLY, ICM implantation ≤30 days after the index event; ICMDELAYED, ICM implantation between 31 and 365 days after the index event.*

**Figure S1. Flow Chart of data.**

**
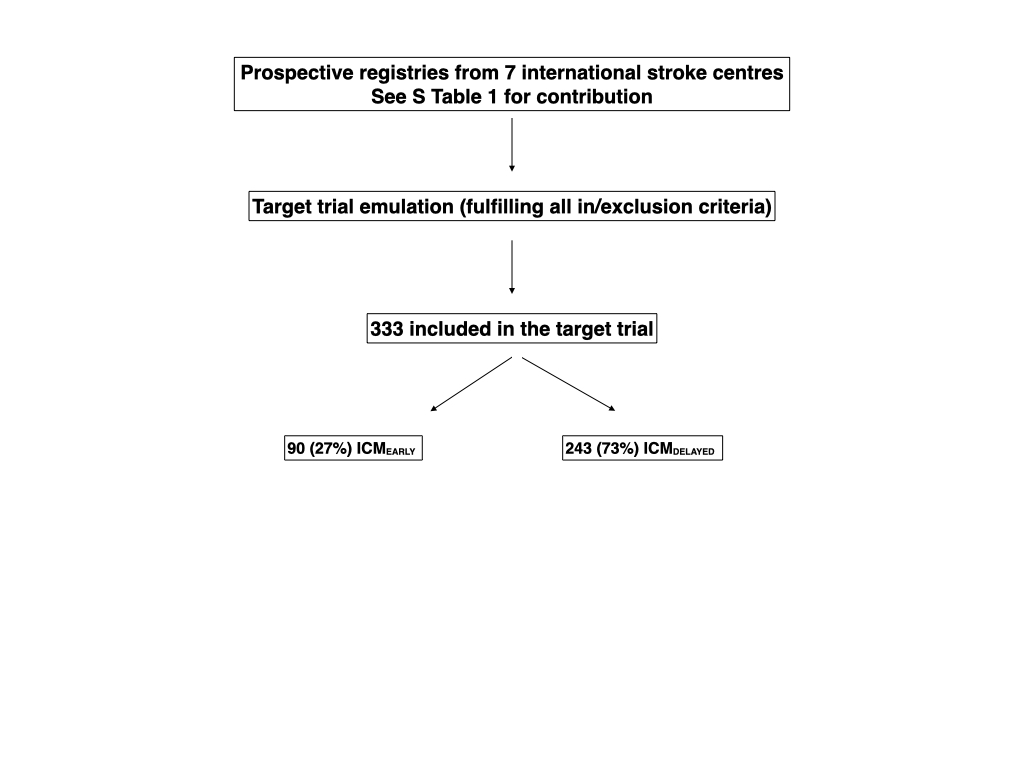
**

**Figure S2. Love plot.**

**
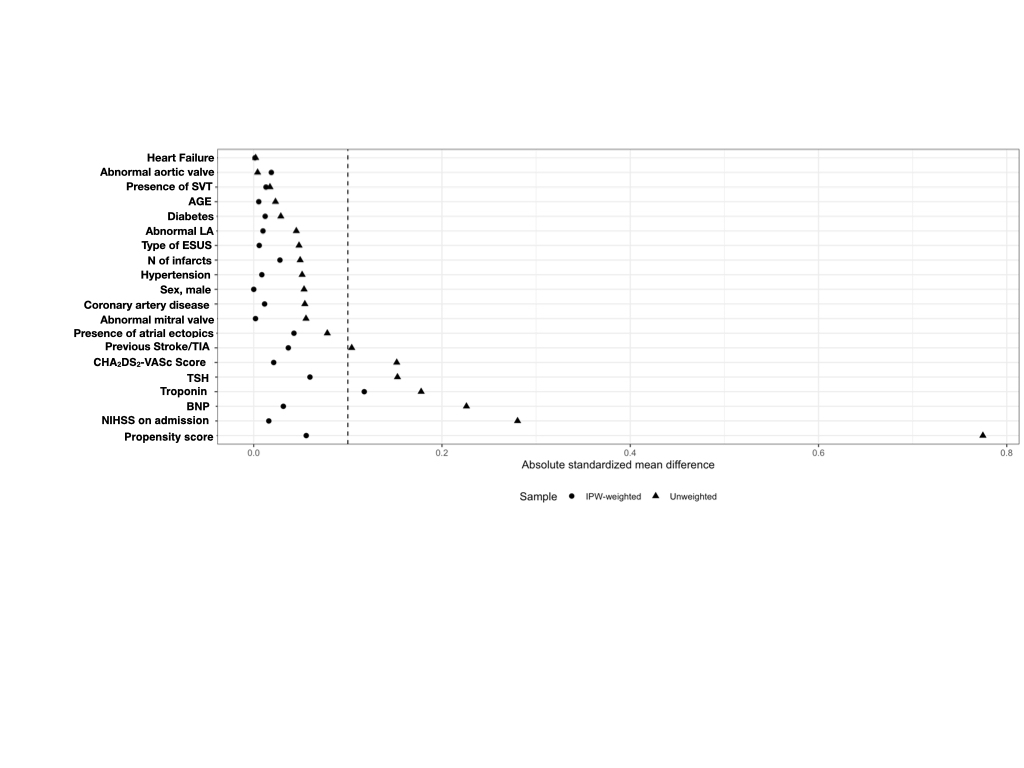
**

**Figure S3. Bar plot of propensity scores distribution stratified by group before and after inverse probability weighting**


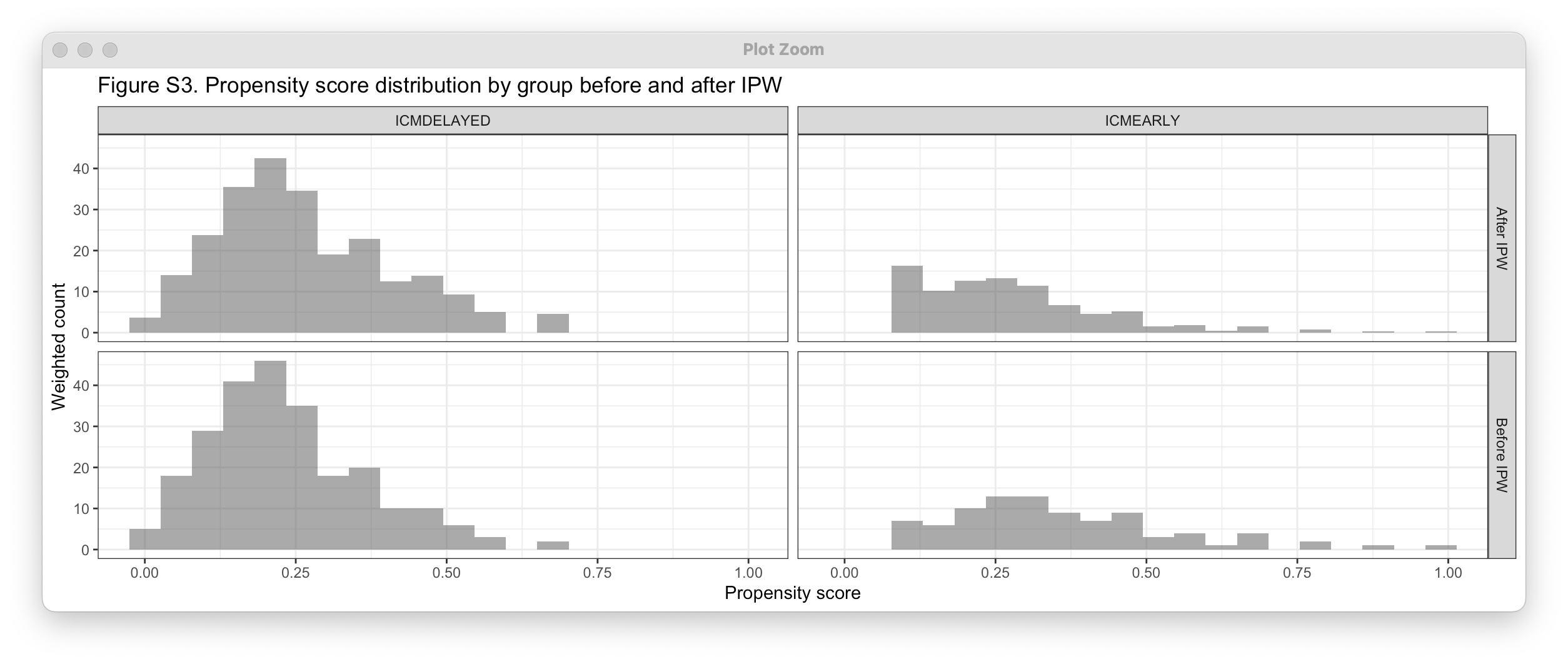

Supplement: sj-docx-2-wso-10.1177_17474930261438742 – Supplemental material for Timing of insertable cardiac monitor implantation after embolic stroke of undetermined source and its impact on atrial fibrillation detection: A target trial emulation analysis [file sj-docx-2-wso-10.1177_17474930261438742.docx]
